# Relationship between SUVmax on 18F-FDG PET and PD-L1 expression in liver metastasis lesions after colon radical operation

**DOI:** 10.1186/s12885-023-11014-x

**Published:** 2023-06-12

**Authors:** Yan Qiao, Xiaomeng Li, Yongquan Hu, Pu Guo, Hengchao Liu, Hong Sun

**Affiliations:** 1 Department of infectious disease, The First Affiliated Hospital of Bengbu Medical College, Anhui, Bengbu, 233004 China; 2 Department of Clinical Laboratory Science, The First Affiliated Hospital of Bengbu Medical College, 287 Zhihuai Rd, Bengbu233004, Bengbu, 233004 China; 3 Department of nuclear medicine, The First Affiliated Hospital of Bengbu Medical College, Anhui, Bengbu, 233004 China

**Keywords:** PD-L1, FDG, Liver metastasis, Colon cancer, PET/CT

## Abstract

**Purpose:**

Our study was to investigate the correlation correlation between FDG uptake and PD-L1 expression of liver metastasis in patients with colon cancer, and to determine the value of FDG-PET in predicting PD-L1 expression in liver metastasis of colon cancer.

**Methods:**

A total of 72 patients with confirmed liver metastasis of colon cancer were included in this retrospective study. The PD-L1 expression and immune cell infiltrating of tumors were determined through immunohistochemistry staining. The SUVmax of liver metastasis lesions were assessed using ^18^ F-FDG PET/CT. The correlation between PD-L1 expression and the clinicopathological were evaluated by the Cox proportional hazards model and the Kaplan-Meier survival analysis.

**Results:**

PD-L1 expression was significantly correlated with FDG uptake (SUVmax), tumor size, differentiation, survival and cytotoxic T cells infiltration in liver metastasis of colon cancer (P < 0.05). And liver metastases with high counts of infiltrating cytotoxic T cells showed greater FDG uptake than those with low counts of infiltrating cytotoxic T cells. The SUVmax of liver metastases and the degree of differentiation of metastases were closely related to PD-L1 expression, and were independent risk factors.The combined assessment of SUVmax values and tthe degree of differentiation of metastase can help determine PD-L1 expression in liver metastasis of colon cancer.

**Conclusions:**

FDG uptake in liver metastasis of colon cancer was positively correlated with the PD-L1 expression and the number of cytotoxic T cells infiltration. The joint evaluation of two parameters, SUVmax and degree of differentiation, can predict PD-L1 expression in liver metastases.

## Introduction

The liver is the main metastatic organ in cases of colon cancer. Liver metastases occur in 50% of the patients with colon cancer after colon radical operation; while 20% of liver metastases can be surgically removed, the remaining 80% cannot be directly removed by surgery [1]. For these patients, traditional chemotherapy or immunotherapy can improve the survive rate and convert liver metastasis to a resectable status [2]. In this regard, anti-programmed death 1 (PD-1)/programmed death ligand 1 (PD-L1) immunotherapy has recently emerged as an effective therapeutic option for liver metastasis of colon cancer [3, 4].

PD1 and PD-L1 are both closely related to immune escape. PD-L1 is mainly expressed on the surface of cancer cells, while PD-1 is the most important ligand of PD-L1. The binding of PD1 to PD-L1 can facilitate immune escape of cancer cells [5]. PD-L1 is expressed in some cancers, including colon cancer, liver cancer, lung cancer, and kidney cancer [6–8]. Anti PD1/PD-L1 immunotherapy shows better treatment efficacy in cancers with high PD-L1 expression. Unfortunately, not all cancer cells show high PD-L1 expression, necessitating assessment of the PD-L1 expression before immunotherapy. Although pathologic diagnosis is the gold standard, it is difficult to assess the PD-L1 expression of every metastatic lesion by multiple biopsies, highlighting the need for a method to dynamically evaluate PD-L1 expression.

Positron emission tomography (PET) imaging can facilitate evaluations of the biological characteristics of tumor [9, 10]. The uptake of fluorodeoxyglucose (FDG) by the primary tumor has been shown to be closely correlated with PD-L1 expression in lung cancer, bladder cancer, and oral cancer [11–13]. However, studies on the correlation between PD-L1 expression and FDG uptake of metastatic lesions are rare, and the relationship between FDG uptake and PD-L1 expression in liver metastatic lesions of colon cancer has not been reported to date.

Therefore, in the present study, we retrospectively analyzed the findings for 72 patients with liver metastasis of colon cancer who had undergone FDG-PET scanning. The PD-L1 expression and infiltration of immune cells in these patients were assessed by immunohistochemical (IHC) analysis. The objective of this study was to evaluate the correlation between FDG uptake and PD-L1 expression of liver metastasis in patients with colon cancer, and to determine the value of FDG-PET in predicting PD-L1 expression in liver metastasis of colon cancer.

## Materials and methods

### Participants

**Fig. 1:**
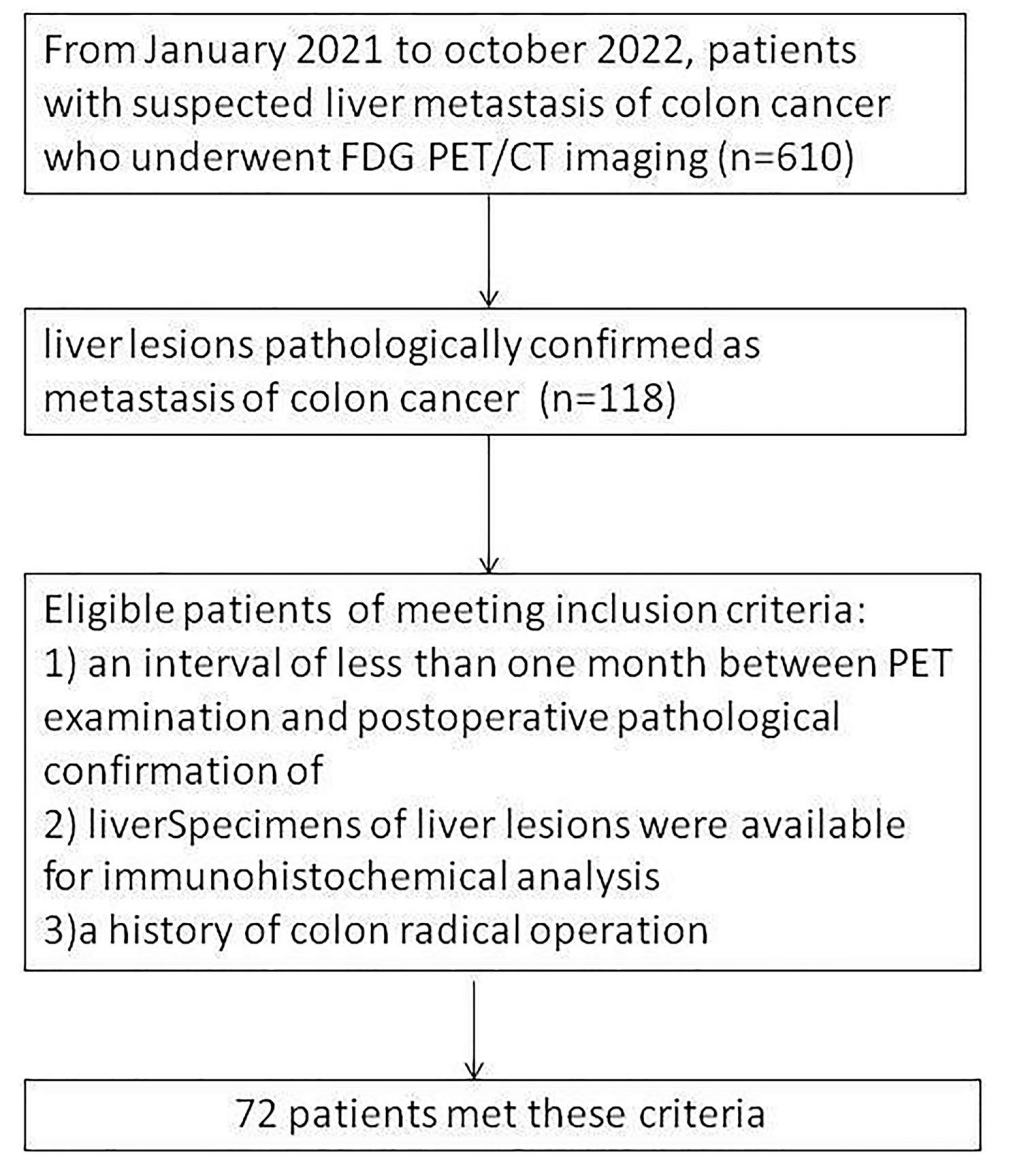
The flowchart shows study inclusion criteria

We retrospectively analyzed 72 patients who showed liver metastasis after colon radical operation and had undergone ^18^F-FDG PET scans at the first affiliated Hospital of Bengbu medical college from January 2021 to October 2022. The inclusion criteria were as follows: 1) liver lesions pathologically confirmed to be metastases of colon cancer; 2) an interval of less than one month between PET examination and postoperative pathological confirmation of liver lesions; and 3) a history of colon radical operation. All 72 patients met these criteria and provided consent for this study (Fig. 1).

### ^18^F-FDG PET/CT examination

All patients were examined using the Biograph 64 PET/CT system (Siemens, Germany). Before the examinations, all patients were required to fast for 4–6 hours and achieve a blood glucose level below 6.3 mmol/L. The patients received an intravenous injection of ^18^F-FDG at 5.55 MBq/kg and underwent PET/CT 60 min after the injection. The CT scanning parameters were as follows: voltage, 120 kV; current, 140 mA. The PET scanning parameters were as follows: 2 min/bed and a 128*128 matrix. PET imaging was performed after CT attenuation correction using ordered subset expectation maximization (OSEM) reconstruction (2 iterations, 28 subsets). PET/CT fusion imaging was acquired using the Siemens post-procession workstation.

### Analysis of images and data

Two experienced nuclear medicine physicians analyzed the PET/CT images. All imaging data were processed using the IntelliSpace workstation (IntelliSpace Portal v7.0; Philips Healthcare, The Netherlands). The lesions were outlined automatically, and the SUVmax was calculated.

### Immunohistochemical staining

The tissue specimens of liver metastasis were embedded in paraffin, sectioned into 4-µm slices, and stained by Nexes automatic immunostainer (Ventana Medical Systems, USA). The primary antibodies against PD-L1, CD8, FOXP3, and CD206 were purchased from Abcam (1:400). The staining intensity was scored as 0–3, and the staining area was scored as 0–3. IHC scores of 0–9 were obtained as the product of staining intensity and area, and IHC scores > 4 were considered to indicate high expression. All results were evaluated by two experienced pathologists [14].

### Tumor microenvironment immune cells and cancer-associated fibroblasts (CAF) counts

CD8, FOXP3, and CD206 are specific markers of cytotoxic T cells, regulatory T cells, M2 macrophages, and respectively [15]. And FAP is a specific marker for cancer associated fibroblasts (CAF). After IHC staining, immune cell counts in the tumor microenvironment were analyzed by Visiopharm software (VISIPPHARM, Denmark). The mean number of cells in three fields was calculated.

### Statistical analysis

The data were analyzed by SPSS 20.0 software. Non-parametric statistical methods were used to test the differences, and P < 0.05 indicated statistical insignificance. The correlation between SUVmax and tumor markers was established by Spearman correlation analyses. Multi-variation method analyzed the independence factors related to PD-L1 expression. ROC analyses were performed for the values of the parameter predicting PD-L1 expression.

## Results

### Relationship between PD-L1 expression and clinicopathologic characteristics in patients with liver metastasis of colon cancer

**Fig. 2:**
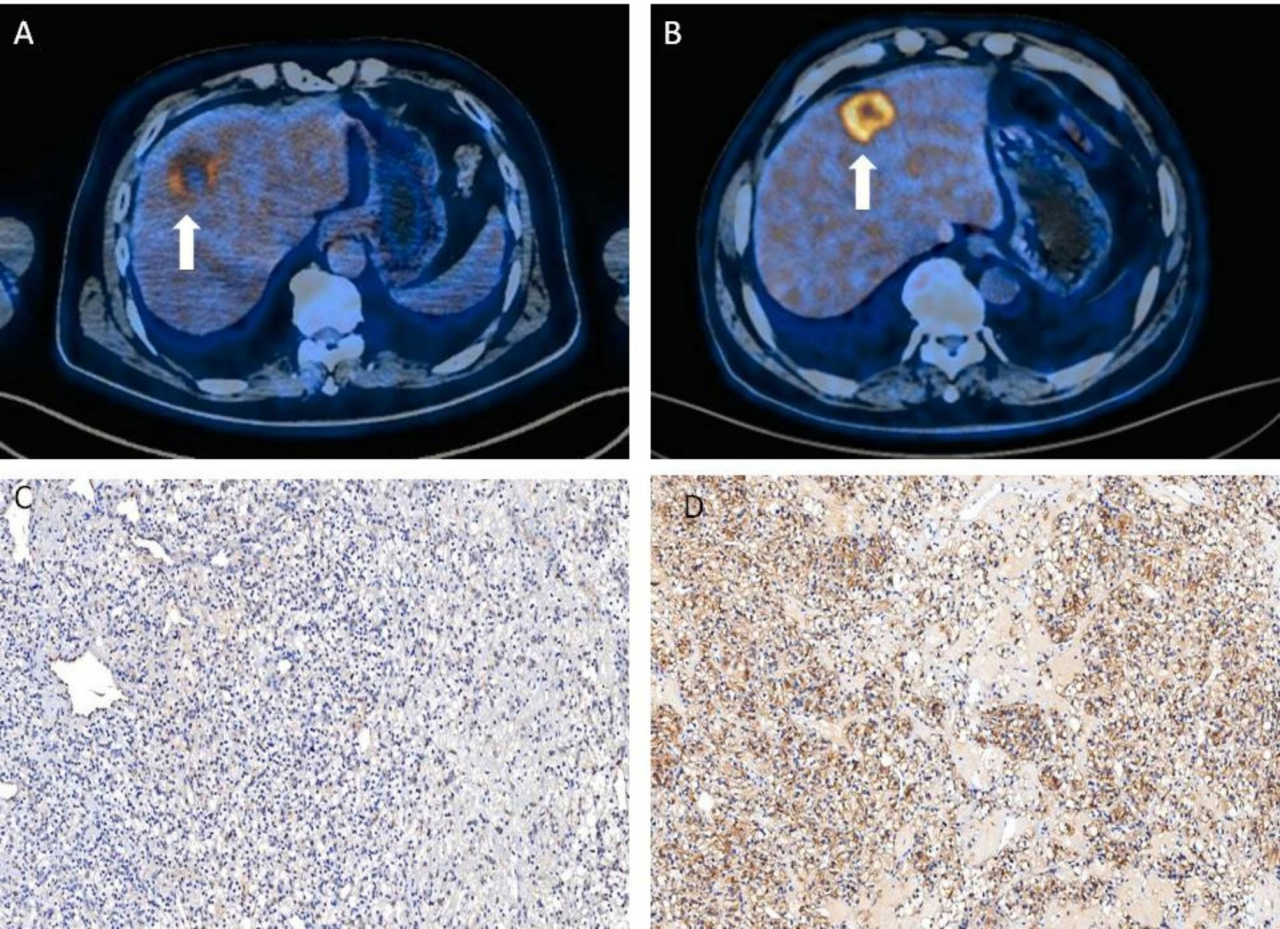
PD-L1 expression is associated with the SUVmax in liver metastasis of colon cancer. (A, C) A 60-year-old male patient showed liver metastasis with negative PD-L1 staining. The tumor lesion did not show obvious ^18^ F-FDG uptake (SUVmax = 3.8). (B, D) A 71-year-old female patient showed liver metastasis with strongly positive PD-L1 staining. The ^18^ F-FDG PET/CT scan showed obvious accumulation of ^18^ F-FDG in the liver lesion (SUVmax = 10.9). Immunohistochemical images were obtained under 400× magnification

A total of 72 patients with liver metastasis (age, 30–75 years; 50 males and 22 females) underwent ^18^F-FDG PET/CT scan before the operation or biopsy. The backgrounds of all patients are shown in Table 1. We first analyzed the correlation between PD-L1 expression in liver metastasis and the clinicopathologic features of the patients. Our results showed that PD-L1 expression is not significantly related to age, sex, other distant metastases and the number of liver metastasis lesions. However, PD-L1 expression was significantly correlated with tumor size, differentiation, and survival. Interestingly, the SUVmax of liver metastasis with high PD-L1 expression was higher than that of liver metastasis showing low PD-L1 expression (P < 0.05; Table 2) (Fig. 2).

**Table 1:** Characteristics of patients with liver metastasis of colon cancer (n = 72)

Variable	N (%)
age	
≥60	36 (50.0%)
< 60	36 (50.0%)
Sex	
male	50 (69.4%)
female	22 (30.6%)
Differentiated	
I-II	40 (55.6%)
III-IV	32 (44.4%)
Tumor size (cm)	
< 3 cm	36 (50.0%)
≥ 3 cm	36 (50.0%)
Liver metastasis	
Single	44 (61.6%)
Multiple	28 (38.4%)
Other distant metastasis	
No	51 (70.8%)
Yes	21 (29.2%)
Survival condition	
Survival	53 (73.6%)
Death	19 (26.4%)

**Table 2:** Relationship between PD-L1 expression and the clinicopathological features of patients with liver metastasis of colon cancer (n = 72)

Clinical variables	PD-L1 (IHC staining)		P-value
	Low expression (n = 49)	High expression (n = 23)	
Age			
≥60 years	23	13	0.614
<60 years	26	10	
Sex			
Male	33	17	0.573
Female	16	6	
Tumor differentiation			
I-II	32	8	0.015
III-IV	17	15	
Tumor size (cm)			
<3 cm	29	7	0.023
≥3 cm	20	16	
Liver metastasis			
Single	32	12	0.287
Multiple	17	11	
Other distant metastasis			
No	36	15	0.647
Yes	13	7	
Survival condition			
Survival	40	13	0.024
Death	9	10	
SUVmax	6.0 ± 2.9	8.5 ± 4.4	< 0.001

### SUVmax values for predicting the expression of PD-L1

**Fig. 3:**
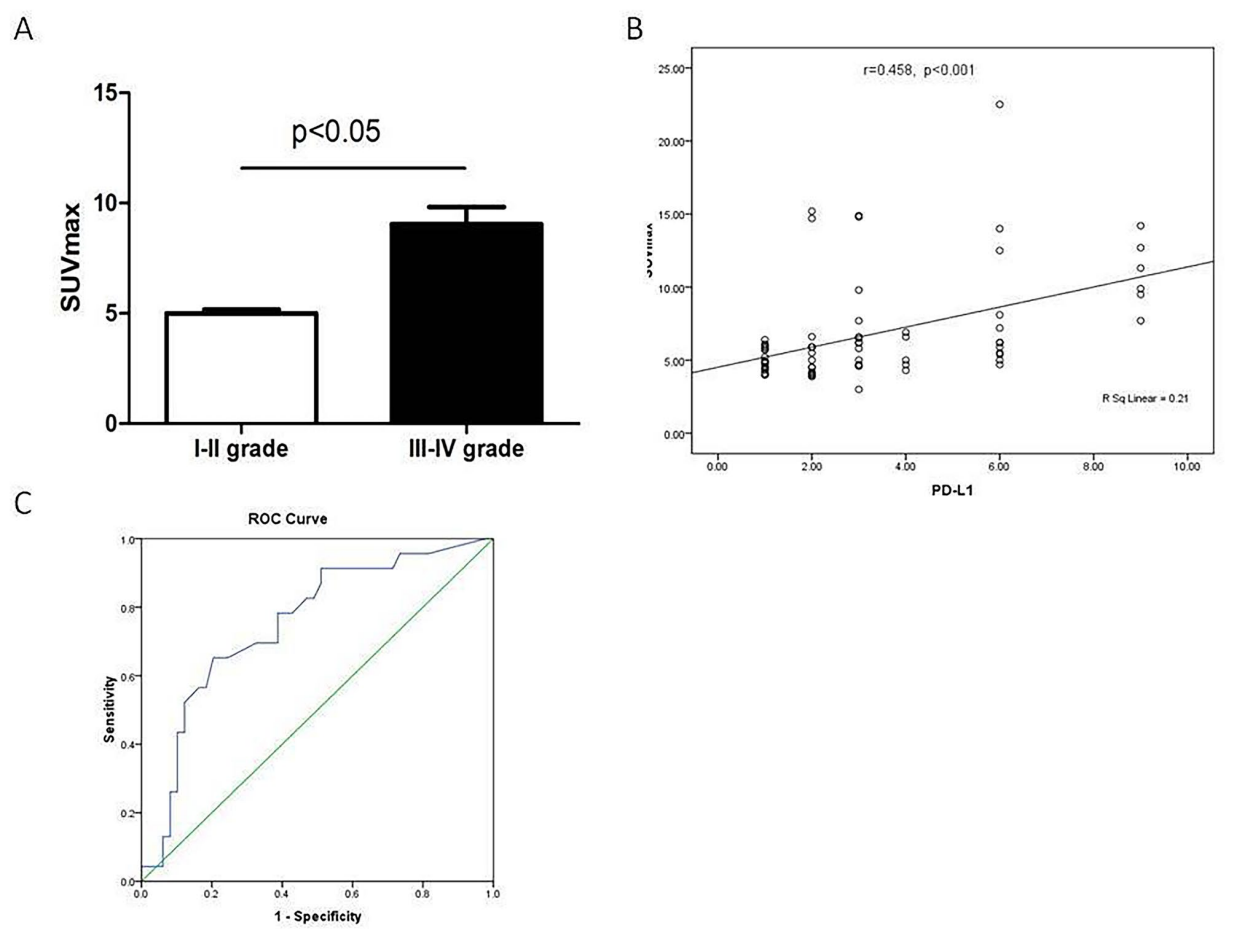
The correlation between SUVmax, tumor differentiation, and PD-L1 expression. (A) The correlation between SUVmax and tumor differentiation. (B) The correlation between SUVmax and the expression of PD-L1. (C) Receiver operator characteristic curve analysis (ROC) of SUVmax for predicting PD-L1 expression in liver metastasis of colon cancer

SUVmax is the most frequently used parameter in analyses based on PET imaging. First, we analyzed the correction between SUVmax and tumor differentiation. As shown in Fig. 3A, the SUVmax of poorly differentiated tumors (III-IV grade) was significantly higher than that of highly differentiated tumors (I-II grade) (P < 0.05). We subsequently attempted to assess the expression of PD-L1 in liver metastasis by ^18^F-FDG PET/CT imaging. Our results showed that the SUVmax of liver metastasis is positively correlated with PD-L1 expression (r = 0.458, p < 0.001; Fig. 3B).

We then evaluated the SUVmax value for predicting PD-L1 expression. The ROC analysis showed that at a SUVmax cut-off value of 6.15, the sensitivity and specificity of predicting PD-L1 expression were 65.2% and 75.5%, respectively (Fig. 3C; Table 3). Table 4 showed the correction between PD-L1 expression and the two groups with a cutoff value of 6.15 for SUVmax. Compared with SUVmax < 6.15 group, the expression level of PD-L1 in SUVmax ≥ 6.15 group was significantly higher (P < 0.001).

**Table 3:** Receiver operating characteristic curves of prediction models for PD-L1 expression

Parameter	AUC	P value	95% CI	Threshold value	Sensitivity	Specificity
SUVmax	0.745	< 0.001	0.626–0.863	6.15	65.2%	75.5%

**Table 4:** PDL-1 high expression between the two groups with a cutoff value of 6.15 for SUVmax (n = 72)

SUVmax	PD-L1 (IHC staining)		P-value
	Low expression (n = 49)	High expression (n = 23)	
≥ 6.15	10	15	< 0.001
< 6.15	39	7	

### The correlation between immune cell infiltration, cancer associated fibroblasts (CAF) distribution in the tumor microenvironment and SUVmax and PD-L1 expression

**Fig. 4:**
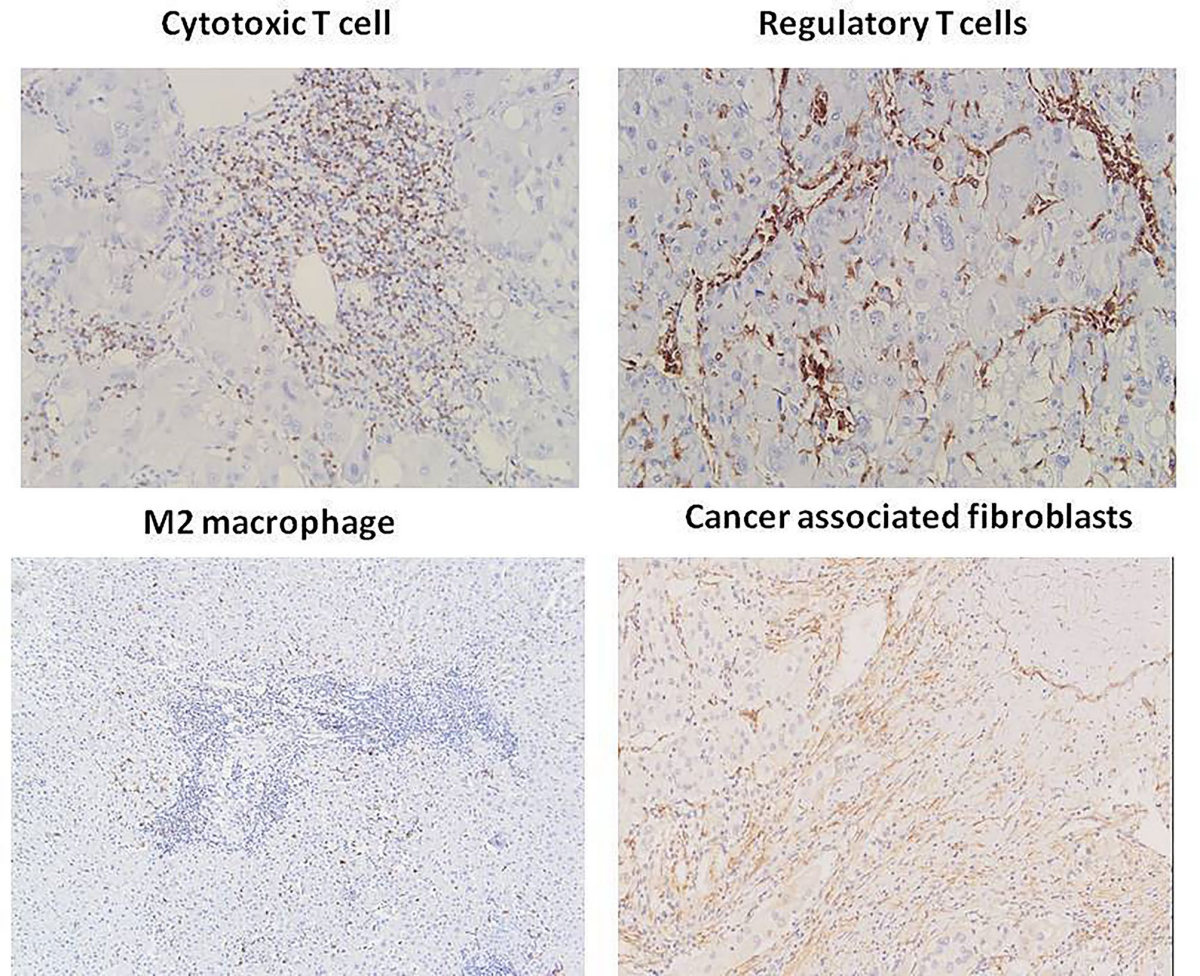
Typical IHC imaging of immune cells and cancer associated fibroblasts infiltrating into liver metastasis of colon cancer. A: infiltrating cytotoxic T cells, B: infiltrating regulatory T cells, C: infiltrating M2 macrophages, D: cancer associated fibroblasts distribution (400x magnification)

Immune cell and CAF infiltration into the tumor microenvironment has been related to the FDG uptake of tumors and anti-PD1/PD-L1 treatment efficacy, but the relevant research on this relationship in liver metastasis is limited. Therefore, we were very interested in the correlation between the immune cell infiltration, CAF *distribution* and the PD-L1 expression and SUVmax in liver metastasis. Cytotoxic T cells, regulatory T cells, M2 macrophages and CAF are the major cells related to immune therapy in the tumor microenvironment. We assessed the counts of infiltrating cytotoxic T cells, regulatory T cells, M2 macrophages and CAF in tumors by IHC (Fig. 4). First, we analyzed the correlation between FDG uptake and immune cells infiltrating liver metastasis. Our results showed that SUVmax is significantly correlated with the number of cytotoxic T cells (r = 0.264, P = 0.025) (Table 5). Liver metastases with high counts of infiltrating cytotoxic T cells showed greater FDG uptake than those with low counts of infiltrating cytotoxic T cells. However, no significant correlation was found between the SUVmax and the number of infiltrating regulatory T cells, M2 macrophages and CAF. Subsequently, we analyzed the correlation between PD-L1 expression and immune cell infiltration in liver metastasis of colon cancer. The results showed that the expression level of PD-L1 was positively correlated with the number of infiltrating cytotoxic T cells (r = 0.350, p = 0.003), but not significantly correlated with the number of infiltrating M2 macrophages, regulatory T cells and CAF (P > 0.05) (Table 6).

**Table 5:** Correlation analysis between ^18^ F-FDG uptake and immune cell, cancer associated fibroblasts count

Factor	SUVmax	
	Correlation coefficient	p-value
Cytotoxic T cells	0.264	0.025
Regulatory T cells	0.001	> 0.05
M2 macrophages	0.088	> 0.05
Cancer associated fibroblasts	0.120	> 0.05

**Table 6:** Correlation analysis between PD-L1 expression and immune cell count

Factor	PD-L1 expression	
	Correlation coefficient	p-value
Cytotoxic T cells	0.350	0.003
Regulator T cells	-0.012	> 0.05
M2 macrophages	-0.040	> 0.05
Cancer associated fibroblasts	0.185	> 0.05

### Multivariate analysis of PD-L1 expression and prediction of PD-L1 expression in liver metastasis

Assessment of the PD-L1 levels in primary tumors and metastases is of important value in evaluating the prognosis of patients and predicting the efficacy of anti-PD1/PD-L1 therapy [16]. Our study showed that PD-L1 expression in colon cancer liver metastases was correlated with SUVmax, tumor differentiation, tumor size, patient survival status, and the number of infiltrating cytotoxic T cells. Therefore, we further explored the independent risk factors affecting PD-L1 expression through multivariate analysis. Our results confirmed that the SUVmax of liver metastases and the degree of differentiation of metastases were closely related to PD-L1 expression, and were independent risk factors (Table 7).

**Table 7:** Multivariate analyses of the predictors of PD-L1 expression in HCC

Variable	Exp (B)	Confidence interval (95%)	*P-*value
SUVmax	18.32	3.40–96.60	0.001
Tumor size	5.04	1.18–22.19	0.061
Tumor differentiation	7.68	1.66–35.49	0.009
Survival status	2.10	0.34–13.11	0.426
Cytotoxic T cells	1.00	0.99–1.01	0.235

Subsequently, we divided liver metastases into groups showing high probability of PD-L1 expression (SUVmax ≥ 6.15 and poor differentiation), medium probability of PD-L1 expression (SUVmax ≥ 6.15 and good differentiation or SUVmax < 6.15 and poor differentiation), and low probability of PD-L1 expression (SUVmax < 4.55 and good differentiation). Our results showed that the probability of predicting high expression of PD-L1 in the high-, medium-, and low-probability groups was 71.4%, 14.7%, and 5.4%, respectively (Table 8).

**Table 8:** Rate of PD-L1 expression in the low-, moderate-, and high-potential groups

Probability	Total (n)	PD-L1 expression		*P-*value
		Low	High	
Low	37	94.6%	5.4%	< 0.001
Moderate	34	85.3%	14.7%	
High	21	28.6%	71.4%	

## Discussion

Tumor immune escape and sensitivity to immunotherapy are current research hotspots. Tumor immune escape and immunotherapy efficacy are closely related to the expression of PD-L1 in tumor cells and the infiltration of inflammatory cells in the tumor microenvironment [17–19]. Thus, assessment of PD-L1 expression will be of great value in guiding immunotherapy. However, studies on the prediction of PD-L1 expression in tumor metastases are relatively scarce. In this study, we focused on the relationship between PD-L1 expression, SUVmax, and inflammatory cell, CAF infiltration in colon cancer liver metastases to explore the correlation between FDG uptake in liver cancer and tumor immunity, and to determine the value of predicting PD-L1 expression by FDG-PET.

In our study, the FDG uptake value of liver metastases, the SUVmax, was closely related to the expression of PD-L1, and liver metastases with high PD-L1 expression usually showed high SUVmax values. Thus, liver cancer with high PD-L1 expression may have high glucose metabolism characteristics, and ^18^F-FDG PET/CT may help evaluate the expression of PD-L1 in patients with liver metastasis to guide immunotherapy. Ruohua Chen et al. [12]also obtained similar results. In their study, higher ^18^F-FDG uptake by bladder cancer was associated with elevated PD-1/PD-L1 expression, and SUVmax value of 22.7 was the best cut-off value for predicting high expression of PD-L1 in bladder cancer. Notably, the correlation mechanism between FDG uptake and PD-L1 protein expression has not been fully elucidated. One possible mechanism is that the key genes regulating glucose metabolism, such as PKM2 and hypoxia factor, regulate the expression of PD-L1 through protein interaction or other signal transduction pathways [20, 21].

Cytotoxic T cells, regulatory T cells, M2 macrophages and CAF are important cells in the tumor microenvironment that regulate tumor immunity [22–24]. Simultaneously, these cells themselves can take up glucose [25], thereby increasing tumor FDG uptake. Therefore, we also aimed to determine the correlation between infiltrating immune cells, CAF and PD-L1 expression in metastases. Our research shows that many liver metastases are accompanied by massive infiltration of cytotoxic T cells, and that the number of infiltrating cells is positively correlated with FDG uptake in metastases. Cytotoxic T cells can specifically recognize peptides on the tumor surface and kill the tumor [26]. There is cytotoxic T cell infiltration in lung cancer, digestive tumor, and so on. Especially in “hot” tumors (sensitive to immunotherapy), there is more cytotoxic T cell infiltration [27]. Thus, ^18^F-FDG PET/CT may be helpful to evaluate the infiltration status of inflammatory cells, especially cytotoxic T cells, in colon cancer liver metastases.

The expression status of tumor PD-L1 is closely related to the efficacy of immunotherapy [28]. To better predict the expression of PD-L1, we further analyzed the correlation between PD-L1 expression and different clinical features. PD-L1 expression was significantly correlated with the degree of tumor differentiation, size, patient survival status, and the number of infiltrating cytotoxic T cells. Multivariate analysis further confirmed that SUVmax and tumor differentiation were independent predictors of PD-L1 expression in liver metastases. On the basis of the results of multivariate analysis, according to the level of SUVmax and the degree of differentiation, we divided liver metastases into high-, medium-, and low-probability groups of PD-L1 expression. In the high-probability group, the predicted probability of high expression of PD-L1 was 71.4%, while the probability of high expression of PD-L1 in the low-probability group was only 5.4%. These results further indicated that ^18^F-FDG PET/CT imaging combined with evaluation of liver cancer SUVmax value and tumor differentiation can help determine the expression of PD-L1 in liver cancer. Nevertheless, this study had some limitations. First, this was a single-center retrospective study. Second, due to the limited number of patients, some validation cohorts are still missing, necessitating prospective validation studies to confirm the current findings.

## Conclusions

In conclusion, the SUVmax and the degree of differentiation of colon cancer liver metastases were found to be independent factors for predicting the expression of PD-L1 in liver metastasis of colon cancer. The joint evaluation of these two parameters, SUVmax and degree of differentiation, can predict PD-L1 expression in liver metastases. We also found that the FDG uptake of liver metastases was positively correlated with the number of infiltrating cytotoxic T cells and that the expression of PD-L1 was also positively correlated with the number of infiltrating cytotoxic T cells. These results can help elucidate the mechanism underlying glucose metabolism and immune evasion of liver cancer, and provide a clinical research basis for the evaluation of tumor immune status by PET/CT.

## Data Availability

The data could be obtained from the corresponding author upon request.
